# Gastrodin and Vascular Dementia: Advances and Current Perspectives

**DOI:** 10.1155/2022/2563934

**Published:** 2022-04-12

**Authors:** Chujun Deng, Huize Chen, Zeyu Meng, Shengxi Meng

**Affiliations:** ^1^Department of Traditional Chinese Medicine, Shanghai Jiao Tong University Affiliated Sixth People's Hospital, Shanghai 200233, China; ^2^The Second Clinical Medical College, Heilongjiang University of Chinese Medicine, Harbin 150040, China

## Abstract

*Gastrodia elata*, a traditional Chinese medicine, has been widely used since ancient times to treat diseases such as dizziness, epilepsy, stroke, and memory loss. Gastrodin, one of the active components of *Gastrodia elata*, has been used in the treatment of migraine, epilepsy, Parkinson's disease, dementia, and depression in recent years. It can improve cognitive function and related neuropsychiatric symptoms through various effects and is considered as a promising treatment for dementia. Vascular dementia is a kind of severe cognitive impairment syndrome caused by vascular factors, and it is the dementia syndrome with the largest number of patients besides Alzheimer's disease. Although there is still a lack of evidence-based explorations, the paper reviewed the mechanism and methods of gastrodin in the treatment of vascular dementia, providing a reference for clinical therapy.

## 1. Introduction

### 1.1. Vascular Dementia

Vascular dementia (VaD) refers to the severe cognitive impairment caused by vascular pathology, that is, the classification of vascular cognitive impairment (VCI) described as severe, which is also the second leading cause of dementia [1]. The pathophysiological mechanism of VaD is mainly that vascular dysfunction causes oxidative stress and inflammation in brain tissue. On the one hand, it leads to a series of subsequent apoptosis and autophagy, resulting in reduced number of neurons and synaptic dysfunction. On the other hand, it causes blood brain barrier damage (BBB), resulting in toxic materials (such as amyloid beta) accumulation, and accelerates the pathological process above. In this process, various factors like damaged blood vessels, impaired functions of microglia and astrocytes, inflammation of the nervous system, and accumulation of toxic substances lead to neuron loss by mutual superposition or cascade reaction and ultimately lead to cognitive deficits [2–4]. The pathology of VaD usually has vascular damage caused by cortical and subcortical microinfarction and neuronal atrophy at the corresponding position besides AD-like pathology. The pathology of patients often has strong heterogeneity, accompanied by pathological changes of other types of dementia. Dementia caused by separate vascular pathology is not common [5–7].

In many cases, the risk markers of VaD are likely to be similar to those of stroke, cerebrovascular disease, and Alzheimer's disease [8–11]. Currently, VaD is mainly treated by targeting vascular diseases and other VaD risk factors, including improving cerebral blood circulation, protecting neurological function, and improving neuropsychiatric symptoms [12]. Although commonly used dementia drugs can show cognitive benefits in some VaD patients, their function and population-wide benefits have remained uncertain [13].

### 1.2. Gastrodin

**Figure 1:**
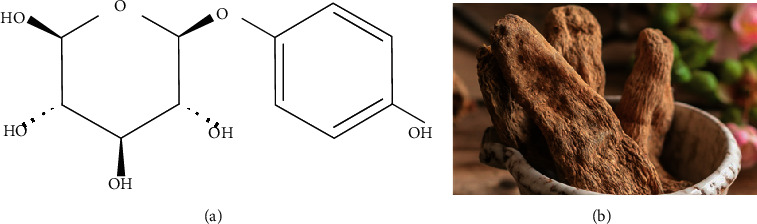
Gastrodin's chemical formula and *Gastrodia elata*.

*Gastrodia elata* has been a traditional medicine for epilepsy, vertigo, headache, numbness, and movement disorders since ancient times (Figure 1(b)). *Gastrodia elata* is composed of many small molecule compounds, such as gastrodin, parishin, phenolic compounds, 4-hydroxybenzyl alcohol, *G. elata* polysaccharides, and *β*-sitosterol. Rhizoma gastrodiae polysaccharides are also one of the main active substances in rhizoma gastrodiae, have a sound neuroprotective effect antiviral, antitumor angiogenesis, and biological activities of osteoporosis, and receive also increased attention from the research community in recent years; however, since the separation of polysaccharide in *Gastrodia elata* in only a few clear the specific structure and function after modification, it is shown that rhizoma gastrodiae polysaccharides research still has a great gap [14].

Here, we focus on gastrodin, another major component of *Gastrodia* that has received much attention in vascular dementia treatment. Gastrodin (GAS), as a phenolic glycoside, and its aglycone (p-hydroxybenzyl alcohol) are major components of the *G. elata* tuber, which are also markers for the quality control and the main active components of this herbal medicine [15] (Figure 1(a)). Gastrodin is naturally found in *Gastrodia elata*, and other *Gastrodia elata* extracts can also be metabolized in the body to produce gastrodin [16].

GAS is separated from *Gastrodia elata* by ethanol extraction. Modern pharmacological studies have found that GAS can be rapidly absorbed through glucose transport channels in the intestinal tract, and the binding rate of GAS and plasma protein is not high [17]. After 15 minutes of intravenous injection, GAS can penetrate the blood-brain barrier (BBB) and can be detected in the frontal lobe, hippocampus, thalamus, and cerebellum of rats, in which the concentration of cerebellum is the highest and excreted through hepatobiliary tract, which has a good effect on improving neurodegenerative diseases. In the central system, it has sedative, neuronal protection, anti-inflammatory, antioxidant, and other therapeutic effects [17–21]. Compared with western medicine in the treatment of VaD, gastrodin has the advantages of fewer side effects, more action sites, and safe efficacy; therefore, it is a promising TCM treatment [22, 23]. In recent years, gastrodin has been developed into a mature and sustainable industrial production mode, which has advantages over traditional Chinese medicine treatment. Gastrodin can play a better role in the treatment of VaD by improving the method of administration, increasing the BBB permeability with the compatibility of other Traditional Chinese medicines, combined with western medicine treatment and risk factor control methods [24–27].

## 2. Materials and Methods

This review covers fifteen years of published literature and focuses on the clinical and experimental studies most relevant to gastrodin therapy for vascular dementia. These include in vitro and in vivo trials of gastrodin in the treatment of VaD and associated risk factors. The mentioned and relevant role and mechanism in these studies have been shown in the figures. All studies were retrieved from the following databases: Web of Science, PubMed, Elsevier, and CNKI. The following keywords were used: “Gastrodin,” “*Gastrodia elata*,” “vascular dementia,” and “Cognitive impairment.” The chemical structures in this article were created by PowerPoint. The figures in this article were created by Adobe Illustrator.

## 3. Results

### 3.1. Role of Gastrodin in the Treatment of Vascular Dementia

*Gastrodia elata* has been used to treat vertebral-basilar artery insufficiency vertigo, neurodegenerative diseases in motility, and cognitive impairment diseases. It was found that gastrodin not only can play a role of neural protection in the early stages of stroke, but also has a good effect on improving vascular cognitive impairment [28].

Gastrodin's neuroprotective role in nervous system is multidimensional. It is well known that microglia play a phagocytic role in the nervous system, and astrocytes can interact with microglia. Both of them jointly regulate inflammatory responses in the nervous system and play a key role in neuroinflammation and degenerative diseases [29]. Any nervous system disorder can lead to inflammation and activation of glial cells, which can lead to neuronal damage and ultimately cognitive deficits after ischemic hypoxia injury. Gastrodin can reduce the inflammatory response and oxidative stress of neuronal cells, regulate the activation of microglia, and reduce the damage of astrocytes, thus reducing neuronal apoptosis [30–32]. The resistance of gastrodin to neurotoxic substances is also a hotspot in recent years. GAS reduces the generation and deposition of A*β* and Tau in cognition-related brain domains and reduces neuroautophagy induced by toxic substances. GAS has been proved to have neural function preservation effect [33, 34]. In addition to reducing the loss of neuron cells, gastrodin's neuroprotective effect is also shown in promoting the secretion of neurotrophic factors, which have been found playing a facilitating role in the survival of neurons and the differentiation of neural stem cells. Therefore, it can be said that its protective effect on nervous system function is bidirectional, and its specific mechanism of neural protection is discussed in the next section of this paper.

Because nerve damage is difficult to reverse, currently accepted treatment strategies for vascular dementia emphasize controlling vascular risk factors for prevention and early control in addition to cognitive improvement. Gastrodin can not only reverse cognitive impairment, it can also ameliorate a number of related risk factors. Many effects of gastrodin have been confirmed, which shows that gastrodin has a promising prospect as an important monomer.

Studies have shown that GAS can effectively intervene in the renin-angiotensin-aldosterone system (RAAS) and its resulting myocardial remodeling, thus playing a role in the treatment of hypertension [35, 36]. In addition to hypertension, atherosclerosis is also a risk factor for cardiovascular and cerebrovascular diseases and for VaD. Gastrodin can restore lysosome function and autophagy activity, thereby reducing lipid accumulation and preventing foam cell formation [37]. In addition to reducing the accumulation of foam cells, GAS can also inhibit the proliferation of vascular smooth muscle cells (VSMC), regulate the effect of vascular smooth muscle, and promote angiogenesis [38–40]. Gastrodin can also improve the intestinal microenvironment in terms of bacterial diversity and abundance, thus reducing inflammation damage to blood vessels and protecting cognitive function through the brain-gut axis [41].

**Figure 2:**
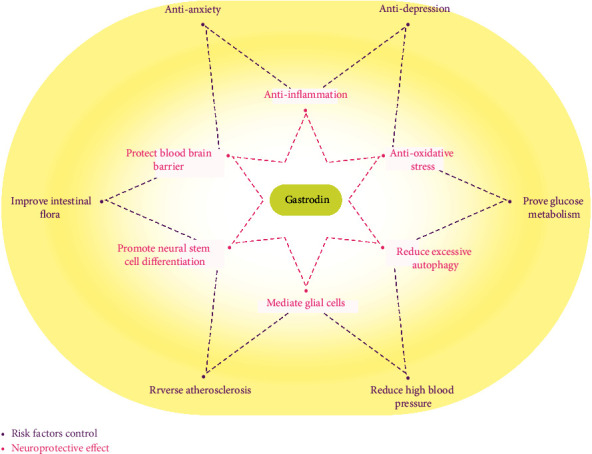
Role of gastrodin in the treatment of vascular dementia.

Although the relationship between depression-like manifestations and vascular dementia is still unclear, various emotional and mental disorders often occur in patients with cerebrovascular disease. Gastrodin has been proved to be effective in the treatment of various mood disorders, especially in the regulation of depression and anxiety related to diabetes and dementia [42–44]. Gastrodin can regulate NLRP3 pathway, inhibit vascular endothelial growth factor, and improve striatum neuron degeneration [45–47]. It has been proved that gastrodin can reduce depression-like behavior by inhibiting ER stress and regulating the neurovascular remodeling associated with Slit Robo A pathway [48–51]. Furthermore, gastrodin also has the regulatory effect of similar to fluoxetine as brain neurotransmitter and improves the anxiety and depression state in the early stage of cognitive impairment by improving the function of monoamine system in the neurotransmission process [52]. Other studies have shown that gastrodin reversed the decrease of *γ* aminobutyric acid (GABA) level and the increase of extracellular glutamate level in the prefrontal cortex and hippocampus of rats with cognitive impairment [53, 54]. The main roles of gastrodin treating vascular dementia have been listed in Figure 2.

Figure 2 pink text shows gastrodin's protective effect on the central nervous system, and purple text shows its effect on metabolic disorders, intestinal flora, and other recently identified risk factors for VaD.

### 3.2. Mechanisms of Gastrodin in the Treatment of Vascular Dementia

Vascular ischemia, toxic substance deposition, and other factors can induce neuroinflammation. When inflammation persists for a long time, the level of oxidative stress in the nervous system will increase, leading to neuronal apoptosis. As the mechanisms of vascular dementia are very diverse, ranging from stroke, cerebral hemorrhage, and vascular disease to vascular dysfunction caused by toxic substances, the research models are also very diverse [55, 56]. Most studies have observed the phenomenon of early onset of inflammatory response and the increase of oxidative stress-related markers. So, reducing mitigating the above threats is a possible treatment for VaD (Table 1).

#### 3.2.1. Gastrodin Reduces the Expression of Inflammatory Factors

**Figure 3:**
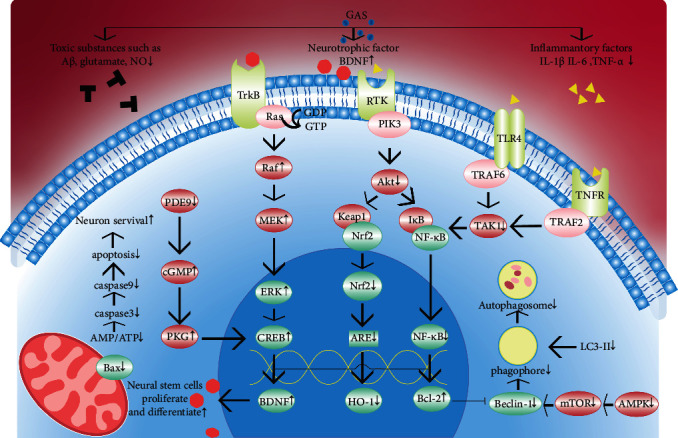
Mechanisms of gastrodin in neurons.

Gastrodin can reduce the levels of inflammatory cytokines TNF-*α*, IL-1*β*, and IL-6 and increase the anti-inflammatory factor IL-10 (Figure 3). Gastrodin can also regulate Mir-21-5p and Mir-331-5p to prevent the inflammatory activation caused by ischemia, thus playing a neuroprotective role [57, 58].

Inhibition of extracellular inflammatory peroxidase (Prx) signaling after stroke appears to be a potential therapeutic strategy [59]. Studies have shown that in vivo metabolites of GAS significantly inhibit macrophages, and Prx1, Prx2, and Prx4 induced inflammation in H_2_O_2_-mediated SH-SY5Y oxidative damage, which can reduce related signal activation of TLR4. It is suggested that TLR4/NF-*κ*B signaling pathway ameliorates neural defects, cerebral infarction, and neuropathological changes in cerebral ischemia-reperfusion rats [60, 61]. What is more, by regulating lncRNA NEAT1/Mir-22-3p axis, gastrodin significantly alleviates the inflammatory response of the nervous system of ischemic rats and alleviates the brain injury in the subacute stage, indicating that gastrodin can play a good therapeutic effect in both early and late pathological changes [62, 63].

Inflammatory factors such as TNF-*α* and IL-1 can bind to the TRAF6 complex and activate its downstream TGF- beta-activated kinase 1 (TAK1). TAK1 is a key regulator of the induction of transcription factor NF-*κ*B and plays a role by mediating I*κ*Bkinase (IKK) activation. Activation of IKK complex results in phosphorylation of I*κ*B-*α*, which is subsequently degraded by the ubiquitin-proteasome pathway. After NF-*κ*B depolymerizes from I*κ*B-*α*, it will be transported into the nucleus to promote NF-*κ*B dependent gene transcription. SSeCKS can promote downstream P38 and Akt signal transduction by promoting TAK1 phosphorylation. Gastrodin inhibits NF-*κ*B signal activation induced by toxic substances and blocks PC12 neuronal apoptosis induced by inflammatory factors by reducing sSECKS-TRAF6 interactions [64] (Figure 3).

#### 3.2.2. Gastrodin Reduces Oxidative Stress Level

Previous studies have found that VaD patients often have elevated levels of oxidative stress in the nervous system, which can lead to inflammation, increase of protease secretion, production of reactive oxygen species, and eventually inducing of neuronal apoptosis. Common antioxidants include superoxide dismutase (SOD), catalase (CAT), and glutathione peroxidase (GSH-PX). Gastrodin has great antioxidant capacity, which can restore the levels of GSH-Px and total mercaptan and reduce the generation of malondialdehyde (MDA) and reactive oxygen species (SOD), thus reversing mitochondrial damage [30, 65]. Gastrodin also activates the p38 mitochondrial protein kinase (P38 MAPK) pathway and reduces the expression levels of P38 and IL-1*β* [66].

At the molecular level, gastrodin upregulates NFE2L2 gene, activates P38 MAPK/Nrf2, and promotes Nrf2 release from the cytoplasmic Kelch-like epichlorohydrin-associated protein 1 (Keap1) and Nrf2 complex. Then, Nrf2 enters the nucleus and binds with antioxidant response elements (ARE), thereby increasing the expression of downstream antioxidant stress factors. One of them is heme oxygenase-1 (HO-1), whose expression has a protective effect on vascular and nerve damage caused by atherosclerosis and ischemia-reperfusion injury [67]. Through this way, GAS can significantly reduce nerve edema after ischemia and improve neuronal apoptosis [68–70]. Gastrodin can also activate Wnt/*β*-catenin signaling pathway through NRF2-mediated antioxidant signals and reduce cycx-2 (COX-2) activation, showing neuroprotective properties [71] (Figure 3).

Through the above pathways, gastrodin significantly increased the expression of Bcl-2 and decreased Bax, thus reducing the levels of active Caspase 3 and active Caspase 9, and played a cognitive protective role, which also proved the role of gastrodin in reducing neuron loss through anti-inflammatory and antioxidant ones [61, 70, 72].

#### 3.2.3. Gastrodin Inhibits Excessive Autophagy Caused by Toxic Substances

Amyloid plaque deposition and Tau phosphorylation are often found in patients with vascular dementia's pathology. Although the mechanism of their role in vascular dementia is still unclear, recent studies have shown that serum A*β*42 is independently associated with cognitive impairment in patients with small vascular disease (CSVD) and can be used to predict cognitive impairment [73]. An overload of neurotoxic substances can cause excessive autophagy, resulting in loss of nerve cells. Common toxic substances, such as A*β*, glutamate, and NO, increase, and neurotrophic substances decrease in VaD patients [74]. Gastrodin can reverse the above pathological changes and play a role in increasing neuronal survival, improving synaptic function and promoting neural differentiation [71]. Studies have shown that GAS can reduce the expression of A*β*1-40/42, APP, and *β*-site APP Exceller enzyme 1. Long-term gastrodin management inhibits the polymerization and decomposition of oligarchic A*β*, prevents its induced cellular neurotoxicity, reduces the level of A*β* plaques and high pTau, and alleviates oxidative stress in APP/PS1 transgenic mice [75] (Figure 3). Excessive autophagy can be inhibited by lowering the levels of Beclin-1, LC3-II, and P62 in both neuronal cells and astrocytes [76, 77]. In addition, the P38 MAPK signaling pathway is also involved in this process [34].

The GAS and its metabolites in the body can effectively reverse A*β* (1–42), causing spontaneous discharge by concentration dependence. This can inhibit the l-type calcium channel current, inhibition of hippocampal neurons M type potassium channel density, and slow down the process of the activation time, alleviate nerve excitability, improve the chain reaction caused by frequent discharge, and reduce cognitive impairment [78, 79].

Gastrodin also has similar effects as an autophagy inhibitor, which can significantly improve the autophagy flux dysfunction of VaD rats by preventing glutamate-induced influx of calcium ions, inhibiting CaMKII activation, inhibiting CaMKII/ASK-1/P38 MAPK/p53 signaling pathway, and increasing lysosomal acidification and autophagosome lysosomal fusion. Eventually, the cognitive defects of vascular dementia rats were reversed [80, 81].

#### 3.2.4. Gastrodin Promotes Neural Survival and Neural Stem Cell Differentiation

Brain-derived neurotrophic factor (BDNF) is mainly expressed in the central nervous system, with the highest content in the hippocampus and cortex. BDNF binds to TrkB to activate the RAS-MAPK pathway and finally activates CAMP response element binding protein (CREB). CREB promotes nerve cell survival, synaptic plasticity, and neurogenesis by increasing the expression of BDNF gene and antiapoptotic protein gene Bcl-2. Gastrodin has been proved to have the ability to repair axons in the peripheral nervous system [82]. In order to illustrate its role in the central system, relevant studies have shown that GAS can promote the reconstruction of peripheral nerve's microvascular network by reducing the expression of TNF-*α* and iNOS. By regulating the cAMP/PKA/CREB signaling pathway, oxidative damage is reduced, and the expression of neurotrophic factor is increased to induce the differentiation of neural stem cells, which has a certain recovery effect on cerebral ischemia focal points [83–85]. Protein kinase G (PKG) is a key downstream effector of cGMP. This pathway promotes the transcription of brain-derived neurotrophic factor (BDNF). PDE9 is specific hydrolase for cGMP, and the hippocampus and cortex are very abundant. Recent studies have found that PDE9 inhibition can promote the proliferation of NSC after oxygen and glucose deprivation injury through the CGMP-PKG pathway [87]. In addition to increasing neuronal survival, gastrodin was observed in animal models to increase the number of neuronal stem cells in the dentate gyrus region, improve cGMP level and upregulate PKG expression by inhibiting PDE9, promote hippocampal neurogenesis after cerebral ischemia, and improve the cognitive function of mice [88–90]. The latest study also showed that it could promote the replication of mouse embryonic neural progenitor cells, further confirming the possible effect of gastrodin on promoting stem cell proliferation and differentiation. GAS targeting SIRT1/BDNF pathway may be a potential therapeutic target for vascular dementia [86].

#### 3.2.5. The Role of Gastrodin in Regulating Glial Cells and Blood-Brain Barrier

Gastrodin regulates microglia-related inflammation in a bidirectional manner. On the one hand, gastrodin promotes microglia to secrete damaging proinflammatory factors that aggravate inflammation. On the other hand, it increases the secretion of protective cytokines by microglia to reduce inflammatory response [91–94].

Gastrodin has been found to inhibit NOTCH-1 signaling pathway in both in vivo and in vitro experiments, and Notch signaling pathway may be involved in the regulation of microglial migration [95]. It reduces the expressions of NICD, RBP-JK, and HES 1, members of the NOTCH-1 signaling pathway, thus enhancing the expression of downstream genes Sirt3 and AT2. And GAS can inhibit the migration of activated BV-2 microglia and inhibit the activation of renin system [70, 96, 97].

Extracellular regulated protein kinases 1 and 2 (ERKl/2) signaling molecules located in the cytoplasm of Ras can be activated by growth factors and enter the nucleus to act as activating transcription factors. Studies have shown that gastrodin not only regulates NRF2-related pathways in neurons, but also inhibits the phosphorylation of ERK1/2, P38 MAPK, Akt, and GSK-3*β* induced by toxic substances by regulating PI3 K/GSK-3*β* signaling pathway in glial cells. Through these pathways, GAS inhibits vascular smooth muscle hyperplasia and alleviates intimal hyperplasia induced by vascular injury [45, 52, 98, 99].

**Figure 4:**
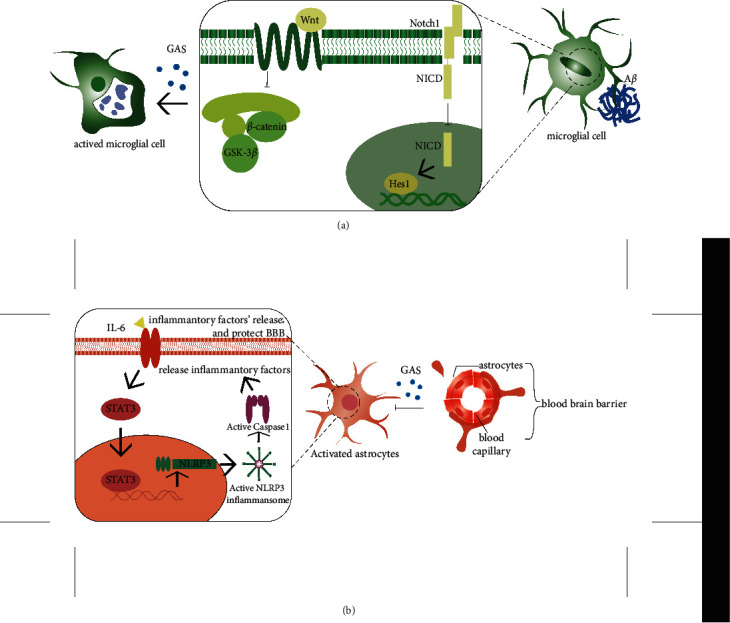
Mechanisms of gastrodin in glial cells.

In addition to the above pathways, gastrodin treatment significantly preserves the Wnt/*β* -catenin signaling pathway in neurons, reduces the activation of cox-2 downstream, promotes neurogenesis, and provides neuroprotection against brain injury [100]. However, gastrodin inhibited the phosphorylation of glycogen synthase kinase-3 *β* (GSK-3*β*) and *β*-catenin induced by lipopolysaccharide in microglia and modulated anti-inflammatory and antiproliferation effects in active microglia [57, 101] (Figure 4(a)).

Transcriptional regulator STAT3 plays an important role in inflammation and natural immune responses, activating NLRP inflammasome and thereby activating the NF-*κ*B pathway. Application of gastrodin in astrocyte models of oxygen and sugar deprivation effectively suppressed inflammation levels in astrocytes and reduced astrocyte loss by inhibiting STAT3 and reducing NF-*κ*B signaling activation [102] (Figure 4(b)).

Many evidences indicate that the cognitive impairment caused by cerebrovascular injury is closely related to the disorder of the blood-brain barrier (BBB). Peripheral toxic A*β* enters the cognition-related brain domains through the impaired BBB and is deposited. Imaging evidence has been observed, suggesting that BBB therapy may be a new therapeutic target for VaD [103–105]. In BV-2 microglia, GAS pretreatment reduced the number of positive cells for matrix metalloproteinase 2 (MMP2) and matrix metalloproteinase 9 (MMP9), two repair agents after nerve injury. Current results suggest that GAS pretreatment significantly compensates for neurobehavioral defects in I/R induced injury rats, reduces the size of cerebral infarction, reverses BBB injury, and alleviates inflammation, which is beneficial during recovery from cerebral ischemia/reperfusion (I/R) injury [90]. Studies have shown that single neurovascular damage caused by HIV infection may be the cause of later cognitive impairment [106]. In the nerve injury model induced by METH and HIV-1 protein, it was found that gastrodin pretreatment significantly increased the expressions of ZO1, JAMA, GLUT1, and GLUT and reversed the blood-brain barrier injury [107] (Figure 4(b)).

### 3.3. Clinical Application of Gastrodin in the Treatment of Vascular Dementia

*Gastrodia elata*'s clinical application and its treatment in cognition-related diseases have been established for a long time. Although the ancients did not have modern diversified ways of drug analysis, through long-term practice, we have found that *Gastrodia elata* combined with other drugs can increase the utilization rate in the nervous system of the active components of *Gastrodia elata*, resulting in better efficacy [108]. In modern times, the ancient prescription is packaged by modern ways, such as Antongshutong capsule. Its protective function of nerve function after stroke has been proved. Prescriptions composed of *Gastrodia elata*, such as Shenma Yizhi Decoction, have been proved to have brain mitochondrial structure improvement effect in VCI rats, thus improving cognitive impairment [109–111]. Of course, better delivery processing of *Gastrodia elata*, such as nasal administration or preparation of *Gastrodia elata* with finer particles, can increase its drug utilization rate. In addition, *Gastrodia elata* can be used with higher efficacy and fewer side effects by modern extraction of gastrodin, the active ingredient in *Gastrodia elata* [112]. Interestingly, gastrodin is hydrophilic, and its combination with porous thin film material polyurethane can promote synaptic generation and provide a good growth matrix, showing its possibility in modern surgical materials exploration [113]. Gastrodin and oxido-uncarine have a synergistic effect to reduce the oxidative stress level of neurons. Oxiracetam combined with gastrodin in the treatment of acute intracerebral hemorrhage can effectively reduce the volume of cerebral hematoma and edema [114]. Gastrodin injection combined with deproteinization of calf serum can effectively improve neurological function score and BDNF level in patients with cerebral infarction [115]. Gastrodin injection combined with hyperbaric oxygen in the treatment of vascular dementia patients can optimize cerebral hemodynamics and improve the level of serum brain-derived neurotrophic factor, which is a very effective combination therapy [116]. Electroacupuncture combined with gastrodin can downregulate the expression of NOGO-A and NgR in the frontal cortex of the ischemic focal area and improve neurological function, which is more effective than electroacupuncture alone or gastrodin [117]. Gastrodin combined with donepezil can improve the quality of life and brain function of patients with vascular dementia, and the effect is better than that of single drug [118]. These methods have shown that gastrodin can improve cerebrovascular and cognition with fewer side effects. And gastrodin can reduce drug dosage when combined with other drugs, showing superior therapeutic effect.

Recent study shows that single pulse of low-energy focused shockwave treatment can get gastrodin through the blood-cerebrospinal fluid barrier [119]. The combination of focused ultrasound (FUS) and microbubbles (MBs) to deliver gastrodin can improve the permeability of its blood-brain barrier (BBB) and improve the drug effect. The combination of gastrodin injection and FUS has also been confirmed to improve cognition in mice with dementia [120, 121]. This indicates that gastrodin does have sufficient cognitive repair function, and its treatment of vascular dementia has realistic scientific basis. Its application method still has great development prospect [122]. Therefore, gastrodin treatment of vascular dementia is a realistic scientific basis, and its application methods still have a great prospect of development.

## 4. Conclusions

Gastrodin has been proved to have anti-inflammatory effects, reduce oxidative stress, reduce nerve apoptosis, regulate nerve autophagy, and have a good neuroprotective effect. Interestingly, many studies have shown that gastrodin can improve anxiety and depression symptoms associated with vascular damage in diabetes and atherosclerosis and dementia. More importantly, gastrodin, a natural extract of the plant *Gastrodia elata*, has better coverage of the complex symptoms of cognitive impairment syndrome than existing dementia drugs and is safer. Therefore, gastrodin can be a promising treatment for vascular dementia due to its wide range of effects. Although gastrodin has a promising future, the BBB penetration rate of gastrodin metabolites is low, and pharmacokinetics suggest that its action time is short. This indicates that gastrodin, as a clinical therapeutic drug, needs to develop more effective drug delivery methods. Other studies have shown that gastrodin in combination with other Chinese or Western medicines can increase the time taken to metabolize the drug and improve cognition. Therefore, the development of diversified drug application methods will be another difficulty in the application of gastrodin. So far, the application of gastrodin lacks sufficient evidence-based evidence, and more clinical studies are needed.

## Figures and Tables

**Table 1:** Mechanism of gastrodin in vivo/vitro models of VaD.

Model	Animal	Cell	Type	Major findings	References
Laparotomy	Mice	—	In vivo	TNF-*α*↓, IL-1*β*↓, IL-6↓, IL-10↑, Prx↓	[58] M. Xiaona, et al 2017
MCAO/H_2_O_2_	Rat	SH-SY5Y	In vivo/In vitro	TLR4↓, NF-*κ*B↓, neuron loss↓, astrocyte and microglia/macrophage activation↓, T-lymphocyte invasion↓, lipid peroxidation↓	[61]Zhang HS, et al 2021
TCDD		C_6_	In vitro	TNF-*α*↓, IL-6↓, iNOS↓, GFAP↓, SSeCKS-TRAF6 interaction↓、NF-*κ*B↓	[62]Jiao M, et al2019
Glutamate	-	HT-22	In vitro	ROS↓, Nrf2 nuclear translocation↑, HO-1↑	[65]Jiang T,et al2020
MCAO	Mice	—	In vivo	Bcl - 2↑, Bax↓, MDA↓ SOD↑, HO-1↑, Nrf2↑	[67]Peng Z,et al 2015
ICH	Rat	—	In vivo	ROS↓, MDA↓, GSH-Px↑, Keap1↑, Nrf2↑, HO-1↑, caspase-3↓, caspase-9↓	[68]Liu X,et al 2020
Pb exposed	Mice	—	In vivo	p-tau↓, A*β*↓, COX-2↓, NR2A, BDNF, Bax↓, Nrf2↑, Wnt/*β*-catenin↑	[69]Liu C-M,et al 2020
APP/PS1/A*β*(1–42)	Rat	SH-SY5Y	In vivo/In vitro	A*β* plaques↓, hyperphosphorylated tau↓, enzymes of A*β*↓, GSK3*β*↓	[74]YQ Zeng,et al2021
MCAO	Rat	—	In vivo	LC3↓, p62↓, phosphorylated CaMKII↓, autophagic flux↓, Ca^2+^↓	[77]Chen T-T,et al2021
Glutamate	—	PC12	In vitro	MMP↓, Ca^2+^ influx↓, CaMKII↓, ASK-1↓, p38 MAPK↓, p53↓、cytochrome C↓,	[78]Jiang G,et al2014
Kainic acid	—	NSC	In vitro	astrocytes↓, neurons↑	[82]Sun G,et al2012
MA	—	PCNC	In vitro	CAMP↑, pPKA↑, pCREB↑, BDNF↑	[83]CL M,et al2020
BCCAO/OGD/R	Mice	HNSCs	In vivo/In vitro	PDE9↓, cGMP-PKG↑, cell viability↑, proliferation in primary hippocampal NSCs↑	[85]H. Xiao,et al2021
A*β* (1–42)	C57BL/6	—	In vivo	SOX-2 and DCX cells in the DG area↑	[86]Li M,et al2016
MCAO	Rat	BV-2	In vivo/In vitro	MMP2↓, MMP9↓, neurological scores↑, the area of cerebral infarction↓	[88]Li S,et al2019
LPS	—	BV-2	In vitro	iNOS↓, COX-2↓, NF-*κ*B↓, CRE-CREB↓, ERK1/2↓, JNK↓, p38MAPK↓	[90]Dai J-N,et al 2011
OGD		BV2	In vitro	Death rate of BV-2 cells↓, several proteins which can affect the MAPK↑	[91]Xia L,et al2014
OGD	—	BV2	In vitro	Inflammatory cytokines↓, protective cytokines↑	[92]Lv Y,et al2021
LPS	Rat	BV-2	In vivo/In vitro	Notch-1↑, NICD↑, RBP-J*κ*↑, Hes-1↑, Notch-1/Sirt3	[93]G. J,et al2021
LPS	Rat	BV-2	In vivo/In vitro	NICD↓, RBP-J*κ*↓, Hes-1↓, ERK, JNK, P38,	[94]Yao Y,et al2021
LPS	Rat	BV-2	In vivo/In vitro	ACE↓, AT1↓, NOX-2↓, iNOS↓, TNF-*α*↓, AT2↑, SIRT3↑	[95]Liu SJ,et al2018
A*β*(1–42)	—	HNC	In vitro	CAT↓, SOD↓, ERKl/2↓	[52]Zhao X,et al2012
MCAO	Mice	—	In vivo	DCX/BrdU double-positive cells↑, infarct volume↓, apoptosis↓, wnt/*β*-catenin↑	[98]Qiu CW,et al2019
LPS	—	BV-2	In vitro	Phosphorylation of GSK-3*β* at ser 9 and *β*-catenin activity↓	[99]Yao Y yi,et al2019
MCAO/OGD	Rat	TNA2	In vivo/In vitro	p-STAT3↓, NLRP3↓, NLRC4↓, caspase-1↓, IL-18↓, NF-*κ*b↓	[100]Sui Y,et al2019

*∗*OGD : Oxygen-glucose-deprivation; *∗* HNC : hippocampus neuronal cell; NNSC : Neural stem cells; PCNC : Primary cortex neuronal cell; HNSC : hippocampal neural stem cell.

## Data Availability

The data used in the current study are included within this article.
